# Supplementary material for: Topological Spin‐Texture Transitions in van der Waals Magnets Revealed by X‐Ray Fourier Transform Holography
Source: Adv Mater. 2026 Sep 8;38(60):e74883. doi: 10.1002/adma.74883 (PMC13712194; doi:10.1002/adma.74883)
Supplement: Supplementary file 1 [file ADMA-38-e74883-s001.docx]

**Supplementary Information**

**Topological spin-texture transitions in van der Waals magnets revealed by X-ray Fourier transform holography**

Sourav Chowdhury¹*^#^, Soumyaranjan Dash^2^*, Michael Schneider^3^, Christopher Klose^3^, Chithra H. Sharma^4,5^, Lisa-Marie Kern^3^, Tim A. Butcher^3,6^, Josefin Fuchs^3^, Santanu Pakhira^7,8^, Samik DuttaGupta^9^, Takashi Taniguchi^10^, Kenji Watanabe^11^, Sujit Das^12^, Sanjeev Kumar^2#^, Bastian Pfau^3^, Amir-Abbas Haghighirad^7#^, & Moritz Hoesch¹^#^

¹Deutsches Elektronen-Synchrotron DESY, Notkestrasse 85, 22607 Hamburg, Germany.

^2^Indian Institute of Science Education and Research (IISER), Mohali, Sector 81, S.A.S. Nagar, 140306, Manauli, India.

^3^Max Born Institute for Nonlinear Optics and Short Pulse Spectroscopy 12489 Berlin, Germany.

^4^Christian-Albrechts-Universität zu Kiel, Leibnizstrasse 19, 24098 Kiel, Germany.

^5^Universität Hamburg, Luruper Chaussee 149, Hamburg 22761 Germany.

^6^Paul Scherrer Institut, 5232 Villigen PSI, Switzerland.

^7^Karlsruhe Institute of Technology, Kaiserstr. 12, D-76131 Karlsruhe, Germany.

^8^Department of Physics, Maulana Azad National Institute of Technology, Bhopal, M.P. - 462003, India.

^9^Saha Institute of Nuclear Physics, 1/AF Bidhannagar, Kolkata 700064, India.

^10^Research Center for Materials Nanoarchitectonics, National Institute for Materials Science, 1-1 Namiki, Tsukuba 305-0044, Japan.

^11^Research Center for Electronic and Optical Materials, National Institute for Materials Science, 1-1 Namiki, Tsukuba 305-0044, Japan.

^12^Materials Research Centre, Indian Institute of Science, 560012 Bengaluru, Karnataka, India.

*Authors contributed equally

#Corresponding authors: (SC) [sourav.chowdhury@desy.de/mailatsourav07@gmail.com](mailto:sourav.chowdhury@desy.de/mailatsourav07@gmail.com), (SK) [sanjeev@iisermohali.ac.in](mailto:sanjeev@iisermohali.ac.in), (A-AH) [amir-abbas.haghighirad@kit.edu](mailto:amir-abbas.haghighirad@kit.edu), (MH) [moritz.hoesch@desy.de](mailto:moritz.hoesch@desy.de)

**Supplementary Note 1:**

**Scaling approach for atomistic simulation of skyrmions.** In atomistic simulations, it is computationally impractical to reproduce the real spatial dimensions of experimental systems. For instance, skyrmions observed experimentally typically have diameters on the order of 100 nm, corresponding to approximately 500 × 500 lattice sites per skyrmion. Simulating a system containing multiple skyrmions would thus require a lattice of about 50,000 × 50,000 sites, far beyond the feasible limit for any Monte Carlo simulation.

To make the problem tractable while retaining the essential physics, we performed simulations on a 120 × 120 lattice and adopted an effectively larger spin-orbit coupling (SOC) strength than that estimated for the real material. The characteristic skyrmion size is known to decrease monotonically with increasing ratio of the SOC strength to the hopping parameter (*λ*/*t*)^1-4^. By increasing *λ*, we reduce the characteristic length scale, enabling the formation of smaller skyrmions compatible with our simulation cell. This scaling approach effectively maps the experimental system onto a reduced computational lattice, reproducing the same qualitative skyrmion textures, stability trends, and field-dependent behavior while maintaining computational feasibility.

**Temperature dependence of model parameters.** All model parameters are expressed in units of the hopping parameter t, which serves as the fundamental energy scale. In double-exchange systems, the electronic bandwidth is known to undergo strong renormalization with temperature. Consequently, if t is assumed to remain constant, the effective values of all other model parameters become temperature-dependent. This temperature dependence plays a crucial role in reproducing the experimental trends observed in our simulations. At the lowest simulated temperature, the parameters are set to *λ* = 0.2, A_u_ = 0.10, and H*_z_* = 0.025, corresponding to an experimental magnetic field of -16 mT. At elevated temperatures, these effective parameter values increase due to the reduction of the electronic bandwidth.

**Discretization effects on skyrmion topology.** For an ideal single skyrmion, the quantity integrated over the lattice area should equal unity. Such definitions assume a continuum vector field describing the spin-texture. In numerical simulations, however, the magnetization is sampled only at discrete lattice points, and the continuous integral is replaced by a finite summation over plaquettes. As a result, the computed value may deviate from an exact integer. For example, an ‘almost perfect’ skyrmion might yield a value of 0.9 instead of 1, while a nearly uniform or topologically trivial configuration may produce a small but finite value, such as 0.05. Therefore, this quantity should be regarded as a quantitative measure of how closely a spin texture approximates a topologically non-trivial configuration, rather than as an exact count. Small deviations from integer values naturally arise due to lattice discretization and slight deformations of the spin-texture. Importantly, the value remains nearly constant as long as the topological character of the spin configuration is preserved, even if its shape or size changes significantly.

**Supplementary Note 2:**

**Effective chiral interactions in a centrosymmetric** **FGT_._** FGT crystallizes in the centrosymmetric space group *P*6_3_/*mmc*^5^, and therefore does not support a global Dzyaloshinskii-Moriya interaction (DMI), which requires broken inversion symmetry. Consequently, the chiral magnetic interactions responsible for noncollinear spin-textures in FGT cannot be attributed to an intrinsic bulk DMI.

In realistic samples, however, inversion symmetry can be broken locally. In exfoliated thin flakes, local inversion symmetry breaking may arise at surfaces and interfaces, as well as from structural inhomogeneities including defects, strain, or stacking variations. While these effects do not imply a globally non-centrosymmetric phase, they can generate effective chiral interactions at the microscopic level.

To capture these effects, we employ a Rashba-type SOC in our theoretical framework. Rashba SOC provides a minimal and generic description of systems where inversion symmetry is locally broken, leading to momentum-dependent spin splitting. In itinerant electron systems with strong Hund’s coupling, such as FGT, the combination of Rashba SOC and electron-spin coupling gives rise to emergent chiral interactions in the effective spin Hamiltonian, including DMI-like terms. In this approach, these interactions are not introduced phenomenologically as an independent DMI term, but instead arise naturally from the underlying electronic Hamiltonian (Eq. (2), Method in the main text).

Band-structure calculations and angle-resolved photoemission spectroscopy (ARPES) studies of FGT reveal strong SOC effects and unconventional band topology^6^. These observations support the relevance of SOC in the low-energy physics of FGT and motivate an effective description that includes SOC-driven chiral interactions. In this context, the Rashba-type SOC used here should be regarded as a minimal effective theoretical construction.

**Role of magnetic inhomogeneities and emergent spin-textures.** The inhomogeneous spin-textures observed in this work (labyrinth domains, isolated skyrmions, and mixed phases) arise dynamically from the competition between exchange interactions, magnetic anisotropy, SOC, dipolar interactions, and external magnetic fields. These inhomogeneities are therefore intrinsic to the energetics of the system, rather than being imposed by quenched disorder.

**Topological characterization via the Bott index.** To characterize the topology of the electronic states in the presence of spatially varying magnetization, we compute the Bott index (See Method in the main text). This quantity is evaluated from the simulated electronic states corresponding to the real-space spin configurations and is known to be equivalent to the Chern number in a magnetically non-periodic system. The Bott index thus provides a measure of the topology of the electronic degrees of freedom in systems with inhomogeneous magnetic textures.

**Supplementary Figures**

**Supplementary Fig. 1| Physical characterization of FGT single crystals: composition and magnetism. a,** Temperature-dependent magnetic volume susceptibility *χ*_V_(*T*) measured during field-cooled warming at *H* = 0.1 T with the field applied along the *ab*-plane (*H* ∥ *ab*) and along the *c*-axis (*H* ∥ *c*). The *T*_C_ ≈ 205 K is consistent with a near-stoichiometric Fe_3_GeTe_2_ composition. **b,** Isothermal magnetization *M*-*H* hysteresis curves at 2 K for *H* ∥ *ab* and *H* ∥ *c*, showing strong uniaxial anisotropy with the *c*-axis as the easy axis. **c,** Surface elemental mapping confirming compositional uniformity. **d,** Display corresponding energy dispersive spectra (EDS) elemental maps over the same region. The individual elemental maps of Fe, Ge, and Te confirm the spatial distribution of each species.

**Supplementary Fig. 2| Sample preparation workflow for synchrotron-based Fourier transform holography (FTH). Upper row:** Schematic illustrates the sequential sample-preparation steps enabling high-spatial-resolution magnetic imaging by FTH. **Lower row:** Corresponding optical micrographs of FGT thin flake at each step, aligned with the schematic above. The FGT flake is encapsulated with ~20 nm of hexagonal boron nitride (*h*BN) to prevent oxidation during handling and measurements. The dotted rectangle displays an SEM image of the final sample configuration, ready for FTH. The object hole (defining the field of view) and the reference holes, both critical for holographic reconstruction, were milled by focused ion beam (FIB).

**Supplementary Fig. 3| Absence of pinning in the spin-texture of FGT.** Spin-textures in a thin FGT flake under applied magnetic fields at 130 K, after repeated field cooling through *T*_C_ with *H_fc_* = 0 mT. Although magnetic nanodomains appear at different locations within the same region of the flake, they consistently exhibit an overall labyrinthine pattern, indicating the absence of strong pinning. The evolution of spin-textures remains qualitatively similar across multiple field-cooling cycles.

**Supplementary Fig. 4| Observation of skyrmion bags in FGT.** Spin-textures in a thin FGT flake under increasing magnetic field *H* at 130 K after field cooling through *T*_C_ at *H_fc_* = -4 mT. The initial spin-configuration at -4 mT is a mixed labyrinth-skyrmion phase. As the field increases, skyrmion bag structures emerge at intermediate fields of 42, 58, 65, and 72 mT. With further field increase, the system transitions to a uniform magnetic domain state, which persists after field reversal.

**Supplementary Fig. 5| Topological transitions and unprotected states near *T*_C_ (194 K).** **a,** Experimental real-space spin-textures in an FGT thin flake during magnetic-field cycling at 194 K after field cooling through *T*_C_ at *H_fc_* = 0 mT. **b,** Theoretical real-space spin-textures from simulations on a *N* = 120² system, after field cooling at *H_Z_* = -0.050 (*T* = 0.07, *λ* = 0.3, *A*_u_ = 0.12). **c,** Variation of skyrmion density *χ*ₛ and Bott index *B* as a function of *H_Z_*, showing a clear topological transition characterized by a zero-line crossing in both quantities. **d,** Schematic depicting topological transitions and the emergence of unprotected states during field cycling.

**Supplementary Fig. 6| Temporal dynamics of spin-textures near *T*_C_ (194 K): a case study of -8 mT data of Fig. 5.** Domain configurations of four sequential time segments (1-4) captured at -8 mT during data acquisition, illustrating time‑resolved evolution and fluctuations of magnetic domain structures in FGT thin flake. These frames highlight the dynamic character of spin-textures near *T*_C_.

**References**

1. P. Sahu, B. R. K. Nanda, S. Satpathy, “Formation of the Skyrmionic Polaron by Rashba and Dresselhaus Spin-Orbit Coupling,” *Physical Review B* 106 (2022): 224403.
2. H. Wu, X. Hu, K. Jing, X. R. Wang, “Size and Profile of Skyrmions in Skyrmion Crystals,” *Communications Physics* 4 (2021): 210.
3. X. R. Wang, X. C. Hu, H. T. Wu, “Stripe Skyrmions and Skyrmion Crystals,” *Communications Physics* 4 (2021): 142.
4. N. Nagaosa, Y. Tokura, “Topological Properties and Dynamics of Magnetic Skyrmions,” *Nature Nanotechnology* 8 (2013): 899-911.
5. H.-J. Deiseroth, K. Aleksandrov, C. Reiner, L. Kienle, R. K. Kremer, “Fe3GeTe2 and Ni3GeTe2 - Two New Layered Transition-Metal Compounds: Crystal Structures, HRTEM Investigations, and Magnetic and Electrical Properties,” *European Journal of Inorganic Chemistry* 2006 (2006): 1561-1567.
6. K. Kim, J. Seo, E. Lee, et al., “Large Anomalous Hall Current Induced by Topological Nodal Lines in a Ferromagnetic van der Waals Semimetal,” *Nature Materials* 17 (2018): 794-799.
